# Effects of Organic Fertilizer Substitution for Chemical Fertilizer Nitrogen and Limited Irrigation on Soil Carbon Emissions in Spring Wheat Fields

**DOI:** 10.3390/plants14213382

**Published:** 2025-11-05

**Authors:** Jun Luo, Min Xie, Zhiwei Zhao, Xiuzhen Ren, Mengyuan Li, Yongping Zhang

**Affiliations:** College of Agronomy, Inner Mongolia Agricultural University, Huhhot 010019, Chinaabc51236@imau.edu.cn (M.L.)

**Keywords:** spring wheat, carbon footprint, saving irrigation, organic fertilizer substitution, chemical fertilizer nitrogen

## Abstract

The Hetao Irrigation District in Inner Mongolia is a major spring wheat production region in China. To synergize high wheat yield, water conservation, and carbon emission reduction in this region, a 2023 and 2024 field experiment was conducted. This study systematically analyzed the effects of organic fertilizer substitution for chemical nitrogen (T1:0%, T2:25%, T3:50%, T4:75%, T5:100%) on soil carbon emissions dynamics and carbon footprint of wheat fields, under two irrigation regimes: water-saving irrigation (twice at jointing and heading stages, 2W) and conventional irrigation (four times at tillering, jointing, heading, and grain-filling stages, 4W). The results showed that during the wheat-growing season, soil CO_2_ emission rate exhibited a single-peak trend (peak at flowering stage), while cumulative soil CO_2_ emission showed a “decrease-increase-decrease” pattern (peak at jointing to heading). At different growth stages, both CO_2_ emission and its rate increased with higher organic fertilizer substitution ratios, and were higher under 4W than 2W. Irrigation and substitution treatments significantly affected the total carbon emissions, carbon sequestration, and carbon footprint: total emissions increased with substitution ratios, while sequestration and footprint first increased then decreased; all three indices were higher under 4W than 2W. Regression analysis revealed that maximum net carbon budget was achieved at 21.6–31.7% substitution (1402.3–1879.9 kg ha^−1^) under 2W, and 31.0–33.8% substitution (2295.5–2822.0 kg ha^−1^) under 4W. In conclusion, water-saving irrigation (900 m^3^ ha^−1^ per application at jointing and heading stages) combined with an optimal organic-nitrogen ratio (1008.0 kg ha^−1^ organic fertilizer, 193.1 kg ha^−1^ chemical nitrogen) effectively coordinates water conservation and carbon emission reduction. This study provides a basis for synergizing these goals in Hetao’s wheat production.

## 1. Introduction

The phenomenon of global warming and climate change, which are the result of the emission of greenhouse gases, such as carbon dioxide (CO_2_), have attracted significant attention on a global scale [1]. The carbon cycle represents a fundamental process of the basic material cycles within terrestrial ecosystems. Soil CO_2_ emissions constitute a significant component of the carbon cycle, and fluctuations in these emissions can directly influence carbon dynamics which, in turn, have substantial implications for global climate change [2]. As a component, soil CO_2_ emissions from agricultural ecosystems have a considerable impact on the global carbon cycle. According to the United Nations Intergovernmental Panel on Climate Change (IPCC), CO_2_ emissions from agricultural soils account for approximately 10~12% of the total global anthropogenic emissions.

Agricultural soil ecosystems are among the most significantly impacted by human intervention. Farm management practices, including tillage, fertilization, and irrigation, interfere with these ecosystems, thereby affecting carbon dioxide emissions [3]. Projections indicate that the ongoing growth in the global population, coupled with an escalating demand for food, will result in a further increase in CO_2_ emissions. This underscores the urgent need to devise effective strategies that balance the maintenance of crop yields with the mitigation of CO_2_ emissions from farmland. The challenge ahead is formidable and will be a pivotal consideration in the future of agriculture [4].

Farm management practices are crucial for minimizing CO_2_ emissions. Various studies have explored the relationship between agricultural practices and greenhouse gas (GHG) emissions, providing insights into effective management strategies [5,6,7] specifically investigated the impact of organic and inorganic fertilizers on carbon sequestration and CO_2_ emissions in a rice–rice cropping system. Their field experiments demonstrated that different treatments, including organic amendments like cow dung and poultry manure, significantly influenced both carbon sequestration and emissions, suggesting that the choice of fertilizer is critical in managing carbon dynamics in agricultural soils. Further research by Wei et al. [8] demonstrated that subsurface watering significantly reduced soil N_2_O and CO_2_ emissions compared to surface watering. This suggests that irrigation management is a critical factor in minimizing GHG emissions from agricultural soils, emphasizing the need for practices that enhance water efficiency while reducing emissions.

Accurate quantification of soil CO_2_ efflux is the premise for assessing soil carbon balance, and the “respiratory approach” has been recognized as a mature and reliable technical system in terrestrial carbon cycle research, with its three mainstream types (alkali absorption method, closed chamber-gas chromatograph method, infra-red gas analyzer method) systematically verified by domestic and international reviews [9,10]. Currently, these approaches have been well applied in different agricultural scenarios: (1) The static closed-chamber method, which estimates respiration rate by measuring CO_2_ concentration changes at fixed intervals, is particularly suitable for regional-scale farmland surveys; (2) The dynamic closed-chamber method, which continuously monitors CO_2_ concentration fluctuations via gas circulation systems [11]; (3) The open-path infrared gas analysis method, which detects CO_2_ concentration differences without chamber closure [12,13], has become the mainstream soil respiration measurement method [14], which is attributed to its three advantages: First, it has a short measurement cycle; second, infrared sensors have high precision; third, portable instruments facilitate field operations. These diversified respiratory measurement approaches provide solid methodological support for accurate quantification of soil CO_2_ efflux in agricultural ecosystems.

Inner Mongolia is the largest and most important spring wheat production area in China, which produces around 32.8% and 23.9% of the total spring wheat area and production in China, respectively. To better understand the effects of field management practices on soil CO_2_ emissions of spring wheat, a field experiment was conducted to investigate the effects of spring wheat field management on soil CO_2_ emissions. We hypothesize that adopting a water-saving irrigation mode reduces carbon emissions by inhibiting soil microbial respiration, while appropriate organic fertilizer substitution ratios enhance carbon sequestration efficiency by increasing wheat biomass. Combining these two practices will achieve the dual effect of “reducing carbon sources and increasing carbon sinks”. The results of this study will provide a feasible technical approach for balancing “carbon emission reduction, and stable grain production” in the spring wheat production of Inner Mongolia.

## 2. Results

### 2.1. Diurnal Changes in Soil CO_2_ Emission Rates

**Figure 1:**
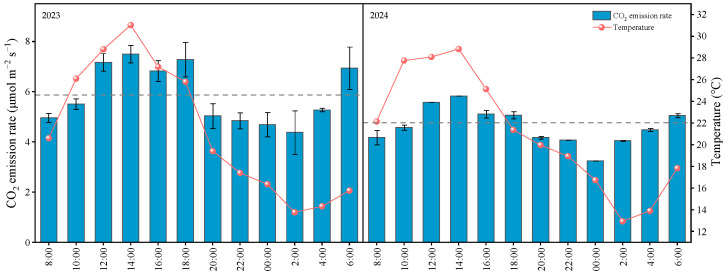
Diurnal variation of soil CO_2_ emission rate during the wheat heading stage. Gray dashed line represents the CO_2_ daily average emission rate.

As shown in Figure 1, the soil CO_2_ emission rates at the heading stage of wheat exhibited obvious diurnal variation patterns in both study years, with a trend of first increasing, then decreasing, and then increasing again—characterized by higher values during the daytime and lower values at night. The ranges of soil CO_2_ emission rates were 4.38–7.50 μmol m^−2^ s^−1^ and 3.25–5.83 μmol m^−2^ s^−1^ in the two years, respectively. The daily maximum and minimum values were observed between 12:00–14:00 and 0:00−2:00, respectively, and the variation trend of soil CO_2_ emission rate was basically synchronized with that of soil temperature. To determine the relationship between soil CO_2_ emission rates at different time periods and the daily average emission rate, the ratio of the emission rate at each time period to the daily average emission rate was calculated. The results showed that the ratios around 10:00 were 0.94 and 0.96 in the two study years, respectively—indicating that the observed values around 10:00 could relatively accurately reflect the daily average soil CO_2_ emission rate. Therefore, when calculating soil carbon emissions at each growth stage of wheat, the observed values at the 10:00 time period can be used as the basis.

### 2.2. Seasonal Changes in Soil CO_2_ Emission Rates

**Figure 2:**
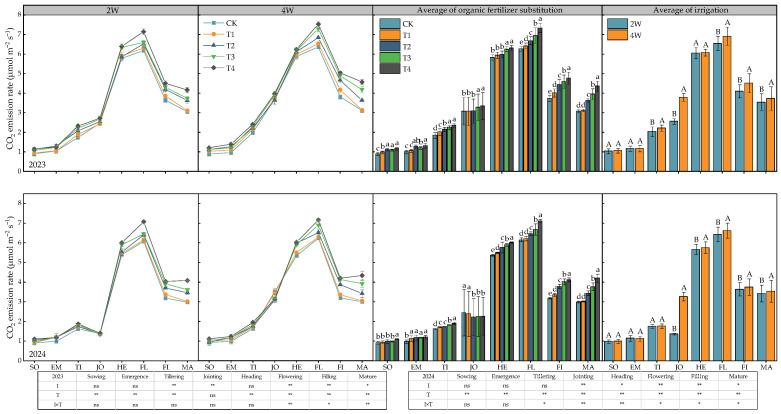
Seasonal changes in soil CO_2_ emission rates under different treatments. SO, EM, TI, JO, HE, FL, FI, MA represents the wheat growing period at sowing, emergence, tilling, jointing, heading, flowering, filling and mature, respectively. Lowercase letters indicate significant differences among fertilization treatments (*p* < 0.05), and uppercase letters indicate significant differences among irrigation treatments (*p* < 0.05). ns indicate no significance at the 0.05 probability level; * and ** indicate significance at the 0.05 and 0.01 probability levels, respectively.

As shown in Figure 2, the soil CO_2_ emission rates under different irrigation and fertilization treatments exhibited obvious seasonal dynamic variation characteristics, presenting a single-peak curve trend of first increasing and then decreasing along with the wheat growth process, and reaching the maximum at the wheat flowering stage. ANOVA indicated that the irrigation regime had a significant or extremely significant effect on the CO_2_ emission rates at each period after the jointing stage. The fertilization treatment had a significant effect on the CO_2_ emission rates throughout the entire growth period. Additionally, the interaction between irrigation and fertilization treatments had a significant effect on the CO_2_ emission rates at each period after the heading stage in 2023 and at each period after seedling emergence in 2024. Among the treatments, under the same fertilization treatment, the CO_2_ emission rates of the 4W (conventional irrigation) treatment after seedling emergence were all higher than those of the 2W (water-saving irrigation) treatment, with the maximum increase amplitude occurring at the jointing stage, reaching more than 47.5%. Under the same irrigation regime, the CO_2_ emission rate increased with the increase in the proportion of organic fertilizer substituting chemical nitrogen fertilizer. The largest difference in CO_2_ emission rates among fertilization treatments was observed at the maturity stage; specifically, the CO_2_ emission rate of the T4 treatment (75% organic fertilizer substitution) was significantly higher than that of the CK (T1, 0% substitution) by 36.2−47.0%.

### 2.3. Differences in Cumulative Soil CO_2_ Emissions

**Figure 3:**
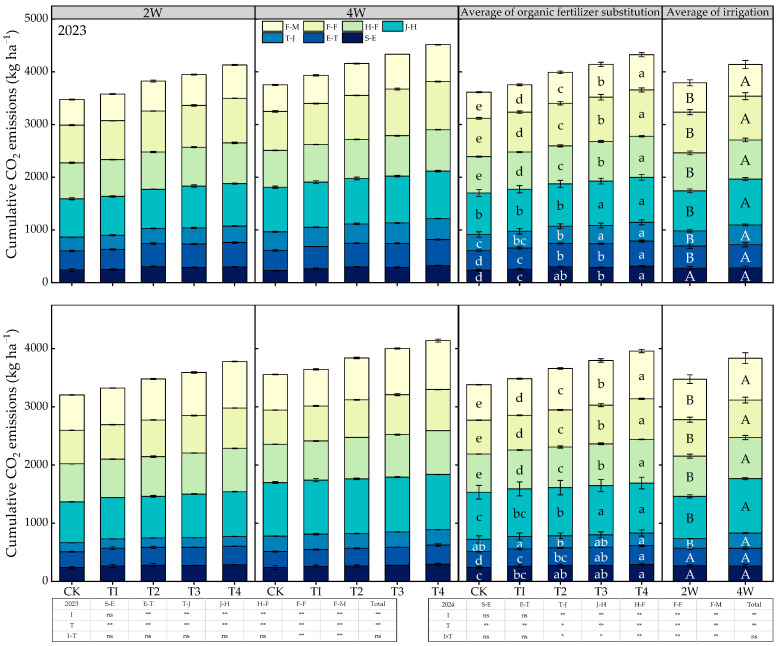
Changes in cumulative CO_2_ emissions at different growth stages under different treatments. S-E, E-T, T-J, J-H, H-F, F-F, F-M represents the growth stage from sowing to emergence, emergence to tillering, tillering to jointing, jointing to heading, heading to flowering, flowering to filling, filling to mature, respectively. Lowercase letters indicate significant differences among fertilization treatments (*p* < 0.05), and up-percase letters indicate significant differences among irrigation treatments (*p* < 0.05). ns indicate no significance at the 0.05 probability level; * and ** indicate significance at the 0.05 and 0.01 probability levels, respectively.

As shown in Figure 3, the cumulative CO_2_ emissions under different treatments exhibited a variation pattern of first decreasing, then increasing, and then decreasing again throughout the entire growing season. The peak values of cumulative CO_2_ emissions were observed at the jointing to heading stage, while the trough values occurred at the tillering to jointing stage. ANOVA indicated that the irrigation regime had an extremely significant effect on the cumulative CO_2_ emissions at the five growth stages after tillering as well as the total cumulative CO_2_ emissions. Under the same fertilization level, the cumulative CO_2_ emissions of the 4W treatment were significantly higher than those of the 2W treatment; the increase amplitudes at the tillering to jointing stage and jointing to heading stage reached more than 30.1% and 14.4%, respectively. The fertilization treatment had an extremely significant effect on the cumulative CO_2_ emissions at all growth stages and the total cumulative CO_2_ emissions. Under the same irrigation regime, the cumulative CO_2_ emissions increased with the increase in the proportion of organic fertilizer substituting chemical nitrogen fertilizer. The differences in cumulative CO_2_ emissions among fertilization treatments were relatively large at the seedling emergence to tillering stage and grain-filling to maturity stage; specifically, the cumulative CO_2_ emissions of the T4 treatment were significantly higher than those of the CK (T1, 0% substitution) by more than 17.1% and 29.3%, respectively, at these two stages. The interaction effect between irrigation and fertilization treatments had a significant effect only on the cumulative CO_2_ emissions at five growth stages (i.e., the flowering to grain-filling stage and grain-filling to maturity stage in 2023, and the tillering to maturity stage in 2024), while it had no significant effect on the total cumulative CO_2_ emissions.

### 2.4. Correlation Between Soil Properties and CO_2_ Emission Rates

As shown in Table 1, there were obvious correlations between different soil properties and soil CO_2_ emission rates at various growth stages, basically showing a positive correlation. After irrigation at the jointing stage, the correlations between soil CO_2_ emission rates and the temperatures of the 10 cm and 20 cm soil layers, as well as the moisture content of the 10 cm soil layer, reached a significant or extremely significant level. In contrast, the correlations between soil CO_2_ emission rates and compactness or soil pH did not reach a significant level. These results indicate that soil temperature and soil moisture content are important factors affecting soil CO_2_ emission rates.

### 2.5. Differences in Carbon Footprint Composition

The carbon footprint of agricultural production refers to the net balance between direct and indirect CO_2_ emissions and sequestration during agricultural production processes, specifically represented as the difference between carbon sinks and carbon sources. As shown in Table 2, the total carbon emissions under different treatments ranged from 6542.3 to 10049.0 kg ha^−1^. Among the sources of these emissions, the carbon emissions from production inputs (including seeds, pesticides, and mechanical fuel) accounted for 4.3−4.8% and 3.5–3.8% under the two irrigation regimes, respectively; emissions from fertilizers (including chemical fertilizers and organic fertilizers) accounted for 19.1% and 15.3%, respectively; emissions from irrigation accounted for 16.9−18.9% and 27.4−29.6%, respectively; and the maximum proportion of soil CO_2_ emissions to total carbon emissions was 57.8% and 51.1%, respectively. Under the same irrigation regime, with the increase in the proportion of organic fertilizer substituting chemical nitrogen fertilizer, the total carbon emissions showed a linear increase, while the total carbon sequestration exhibited a single-peak curve pattern. The total carbon sequestration reached the maximum value in the T1 treatment, which was significantly different from other treatments and significantly higher than that of CK (T1, 0% substitution) by 11.7−23.2%. Under the same fertilization level, compared with the 2W (water-saving irrigation) treatment, the total carbon emissions and total carbon sequestration of the 4W (conventional irrigation) treatment were significantly increased by 22.4−25.1% and 0.9−6.5%, respectively. These results indicate that appropriate irrigation and increased application of organic fertilizers are beneficial to enhancing total carbon sequestration.

### 2.6. Relationship Between NEP and Substitution

**Figure 4:**
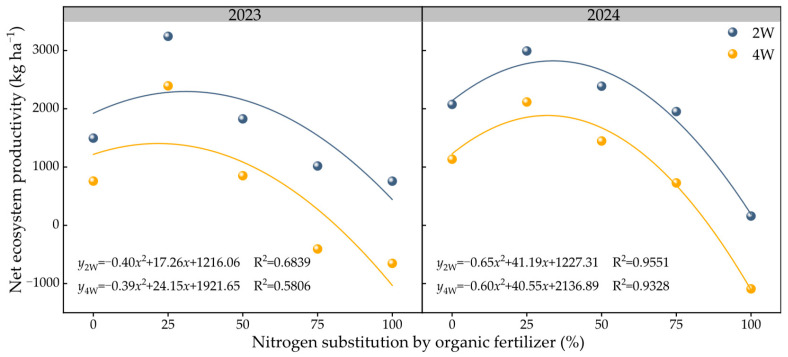
Changes in cumulative CO_2_ emissions at different growth stages under different treatments. S-E, E-T, T-J, J-H, H-F, F-F, F-M represents the growth stage from sowing to emergence, emergence to tillering, tillering to jointing, jointing to heading, heading to flowering, flowering to filling, filling to mature, respectively.

As shown in Figure 4, during the two-year experiment, all treatments except the 4WT3 and 4WT4 treatments exhibited net carbon sequestration characteristics, indicating that the experimental wheat field system generally functioned as a carbon sink. The carbon footprint of different treatments showed that the net carbon budget increased with the increase in irrigation amount, while it presented a trend of first increasing and then decreasing with the increase in the proportion of organic fertilizer substituting chemical nitrogen fertilizer. This indicates that within a certain range, organic fertilizer substitution treatment, although increasing carbon emissions in wheat fields, resulted in a reduced carbon footprint due to the significant increase in total carbon sequestration by wheat, thereby enhancing the carbon sequestration capacity of the wheat field system. Under the two irrigation regimes, linear regression analysis (binary first-order fitting) was conducted between the net carbon budget (y) and the proportion of organic fertilizer substituting chemical nitrogen fertilizer (x). The R^2^ values of the regression equations were all above 0.58, exhibiting good fitting degrees. The maximum values of each regression equation and the corresponding proportions of organic fertilizer substitution were calculated, respectively. Under the water-saving irrigation regime (2W), the maximum net carbon budget (1402.3−1879.9 kg ha^−1^) was achieved when the proportion of organic fertilizer substitution ranged from 21.6% to 31.7%. Under the conventional irrigation regime (4W), the maximum net carbon budget (2295.5−2822.0 kg ha⁻¹) was obtained when the proportion of organic fertilizer substitution was between 31.0% and 33.8%.

## 3. Discussion

Irrigation and fertilization treatments are two crucial factors affecting soil CO_2_ emissions, exerting effects on soil physicochemical properties, which in turn influences microbial activity and the decomposition of soil organic matter, and ultimately exerts a significant impact on soil CO_2_ emissions and carbon footprint in wheat fields [15,16,17]. Throughout the entire growth cycle of wheat, soil carbon emissions exhibit distinct dynamic changes [18,19]. Irrigation directly affects soil CO_2_ emissions: appropriate irrigation can provide favorable environmental conditions for soil microorganisms, promote microbial activity and the decomposition of organic matter, thereby increasing soil respiration. In contrast, excessive or insufficient irrigation may inhibit soil respiration [20,21]. An appropriate proportion of organic fertilizer substituting chemical nitrogen fertilizer not only improves soil structure but also provides abundant carbon sources for soil microorganisms, promoting microbial growth and metabolism as well as enhancing microbial activity, which consequently stimulates soil respiration. Results of this study indicate that under water-saving irrigation (2W), the soil CO_2_ emission rate was significantly lower than that under conventional irrigation (4W). With the increase in the proportion of organic fertilizer substitution, the soil CO_2_ emission rate first increased and then decreased, among which the T1 treatment (0% organic fertilizer substitution) had the highest CO_2_ emission rate. Soil CO_2_ emissions are not only regulated by water and fertilizer management measures but also closely related to the dynamic characteristics of crops at different growth stages. Specifically, there is synergy and consistency between the intensity of soil respiration and crop growth, and the variation in the intensity of soil respiration shows a regular response to the physiological activities of plants [22,23].

Carbon footprint refers to the total cumulative carbon emissions (direct or indirect) of crops throughout their entire life cycle [24,25]. For the entire growth period of wheat, this includes carbon emissions generated from activities such as energy consumption, irrigation, and fertilizer use during the production process. The analysis of carbon footprint requires a comprehensive consideration of the relationship between greenhouse gas emissions and crop production efficiency; subsequent analysis of the impact of agricultural carbon footprint on global climate change can thereby better facilitate the achievement of the goal of stable yield and emission reduction in agricultural systems [26]. Different irrigation and fertilization treatments can alter the energy input during wheat production, thereby exerting a significant impact on carbon footprint [27,28]. Appropriate irrigation can promote the growth and development of plant roots, increase the input of biochar, and enhance microbial activity, thus improving the capacity of soil organic carbon sequestration. In contrast, excessive irrigation may lead to issues such as soil erosion and nutrient loss, which in turn increase the carbon footprint [29]. Studies have shown that water-saving irrigation can reduce the frequency of irrigation, optimize soil moisture conditions, and lower energy consumption, thereby decreasing soil respiration intensity and carbon emissions. Compared with conventional irrigation, water-saving irrigation can significantly reduce the carbon footprint per unit yield [30]. An appropriate amount of organic fertilizer substituting chemical fertilizer can reduce the application rate of chemical fertilizer while improving the utilization efficiency of chemical fertilizer, thereby reducing soil carbon emissions to a certain extent [31,32]. This indicates that the interaction between irrigation and fertilization will further affect the efficiency of the carbon cycle. The results of this experiment show that with the increase in the proportion of organic fertilizer substitution, the carbon footprint first increased and then decreased, with the T1 treatment showing the lowest carbon footprint under both irrigation regimes. Compared with the water-saving irrigation regime, although conventional irrigation can increase the biomass accumulation of wheat, it significantly increases CO_2_ emissions per unit area and consequently raises the carbon footprint. Therefore, only by optimizing the coordinated management of water and fertilizer can the goal of high yield and emission reduction be achieved.

## 4. Materials and Methods

### 4.1. Experiment Site

**Figure 5:**
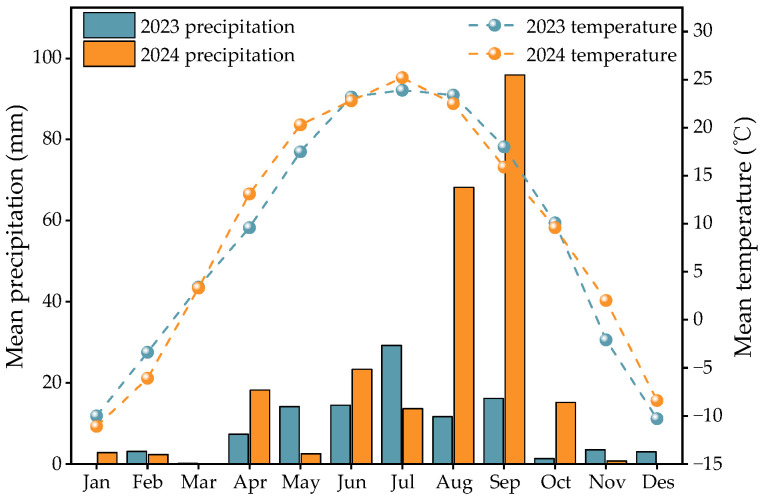
Evolution of monthly precipitation and temperature for 2023 and 2024.

The experiment was conducted from March to August each year on the experimental field of XinGongzhong Town, Wuyuan County, Bayannur City, Inner Mongolia (41°04′ N, 108°03′ E; altitude 1028 m). The monthly precipitation and air temperature during 2023 and 2024 were showed in Figure 5. The experimental field soils texture is sandy soil, classified as endosalic cumulic anthrosols according to the World Reference Base for Soil Resources (WRB, 2014; 2022), corresponding to “saline irrigation-silted soil” in the Chinese soil taxonomy, exhibited organic matter of the 0~20 cm tillage layer was 19.02 g kg^−1^, alkaline dissolved nitrogen 42.04 mg kg^−1^, quick phosphorus 21.92 mg kg^−1^, quick potassium 99.31 mg kg^−1^, and pH = 7.98.

### 4.2. Experiment Design

The experimental study was conducted using wheat cultivar *Yongliang 4* during 2023 and 2024, employing a split-plot design with irrigation regime as the main plot factor and organic fertilizer substitution as the subplot factor. Two irrigation treatments were implemented: water-saving irrigation (2W) involving two irrigations at jointing and heading stages (900 m^3^ ha^−1^ per irrigation), and conventional irrigation (4W) with four irrigations at tillering, jointing, heading, and grain-filling stages (same water amount per irrigation). Fertilization shown in Table 3 included five treatment levels: conventional farmer practice (CK); 25% nitrogen substitution (T1); 50% nitrogen substitution (T2); 75% nitrogen substitution (T3); and 100% nitrogen substitution (T4). Each treatment was replicated three times, resulting in a total of 30 plots, each with an area of 36 m^2^ (4 m × 9 m). Chemical fertilizers included urea and diammonium phosphate, while the organic fertilizer was a bio-organic fertilizer (organic matter ≥ 45%, N + P_2_O_5_ + K_2_O ≥ 7%, and beneficial bacteria ≥ 200 million g^−1^). In the fertilizer management strategy, diammonium phosphate and organic fertilizer were entirely applied as base fertilizer during sowing, while urea was entirely applied as topdressing and evenly broadcast on the soil surface before irrigation during wheat tillering to jointing stage. Wheat was sown using a mechanical seeder with a row spacing of 15 cm and a seeding rate of 412.5 kg ha^−1^. Other management practices followed conventional wheat cultivation.

### 4.3. Data Collection

#### 4.3.1. Soil Respiration Measurements and Analysis

In each plot, measurements of field soil respiration were conducted with SRS-SD1000 (ADC Bioscientific Ltd., Hoddesdon, UK)—a portable soil respiration system—whose respiration chamber features a detachable double-layer structure: a stainless-steel base (lower layer) and a transparent polypropylene cover (upper layer). With a total volume of 1 L and a diameter of 130 mm, the chamber is equipped with a miniature infrared gas analyzer (IRGA) and a miniature air pump installed inside the top of its upper cover, while a soil temperature sensor is embedded in the outer side of the cover. It is characterized by its compact and lightweight design, making it suitable for field work [33,34].

Prior to soil respiration measurements, stainless steel bases were pre-embedded each plot 1 day in advance to eliminate the impact of soil disturbance. Before on-site measurement, the instrument host was powered on and preheated for 20 min to reach the operating temperature of the infrared gas analyzer (IRGA). During measurement for each plot, the transparent chamber cover was first tightly fastened to the pre-embedded stainless-steel base, then the air pump was activated. After 10 min, collecting data on CO_2_ concentration and soil temperature for 2 min (the first 30 s as stabilization period and the subsequent 90 s as the valid data segment), and automatically calculating the soil respiration rate. To ensure data reliability, each plot was measured consecutively 4 times (with a 1 min interval). To minimize measurement errors, readings are taken between 9:00 and 11:00 at the same time of day. Measurements were not taken during or shortly after precipitation. In the experiment, measurements were taken at the sowing, emergence, tillering, heading, anthesis, grain filling, and maturity stages, under clear or partly cloudy conditions.

In addition, to understand the relationship between soil CO_2_ emission rates at different time periods on the same day and the daily average soil CO_2_ emission rate, and thus select a measurement period that best reflects the daily average soil CO_2_ emission rate, we selected plot 4WT1 and conducted diurnal soil respiration rate measurements during the grain filling period (June 22). Starting at 8:00 a.m., soil respiration rates were measured every 2 h, with 10 measurements repeated at each time point, each time interval being 10 s. Soil temperature and water content in the 10 cm soil layer was also measured simultaneously.

#### 4.3.2. Determination of Soil Properties

Soil temperature and soil moisture were measured using a soil temperature meter and a soil moisture meter (Top Instruments Agricultural Environment Detector T30, Zhejiang, China), respectively. Soil compaction was measured using a soil compaction meter (Hengmei HM-JSD.2, Shandong, China). Soil pH was measured using the potentiometric method after standing the soil. The measurements were repeated five times in each plot.

### 4.4. Data Calculation

#### 4.4.1. Cumulative Soil CO_2_ Emissions

According to the calculation of reference [35,36], the formula for soil CO_2_ cumulative emissions is as follows:

(1) $$\sum \frac{Rs_{i+1}+Rs_{i}}{2}\times 86\text{,}400\times 12\times 10^{-6}\times n$$

where *Rs* is the soil respiration intensity (μmol m^−2^ s^−1^); *i* + 1 and *i* represent the time between two subsequent measurements, *n* represents the interval between two adjacent measurements (d); 86,400 represents the conversion of seconds to days; 12 is the molar mass of C; 10^−6^ represents the conversion of μg m^−2^ to kg ha^−1^.

#### 4.4.2. Carbon Footprint of Wheat Production

According to the calculation of reference [37], the calculations of carbon absorption (CA), carbon emissions from agricultural inputs (CF), total carbon emissions (CT), and carbon budget balance are conducted (NEP). CA refers to the amount of carbon sequestered by crops from the atmosphere through photosynthesis. CA was estimated based on the carbon content in the aboveground parts of wheat, the formula for calculating CA is as follows:

(2) CA = DW × Cf

where DW represents the biological yield of wheat, Cf denotes the carbon absorption efficiency of wheat, which is approximately 0.45 g of carbon to synthesize 1 g of organic matter.

The formula for calculating total carbon emissions (CT) is as follows:

(3) CT = CF + CS

where CF is carbon emissions from farmland inputs; and CS is carbon emissions from wheat fields. Wheat field carbon emissions are estimated using soil CO_2_ respiration data.

Farmland input carbon emissions (CF) include direct or indirect carbon releases from inputs such as pesticides, agricultural machinery, irrigation, seeds, and fertilizers during production, transportation, and use. The calculation formula is:

(4) CF = ∑ (Ai × EF_j_)

where Ai is the total amount of each agricultural input (e.g., kg of fertilizer or pesticide, kWh of electricity); EF_j_ is the emission parameter. In calculating the carbon cost, energy consumption for sowing and tillage is represented by diesel consumption, and energy consumption for irrigation is represented by electricity consumption (Table 4).

This study uses the carbon footprint method to assess the carbon balance of wheat field ecosystems. The wheat field carbon budget (NEP) is calculated as follows:

(5) NEP = CT − CA

where CT is the total carbon emissions, and CA is the carbon absorption.

### 4.5. Data Statistical Analysis

The results were subsequently subjected to statistical analysis. The assumption of normality was verified using the Shapiro–Wilk test, with results of all datasets showing *p* > 0.05, indicating no significant departure from normality and thus meeting the assumptions for subsequent parametric analyses. A split-plot analysis of variance (ANOVA) model was performed at 5% probability level, followed by HSD Tukey test. Pearson’s correlation coefficients between the traits were subsequently calculated. All data were performed using SAS 9.0 software (SAS, Cary, NC, USA). Origin 2021 software (Origin LAB, Northampton, MA, USA) was used for graphing.

## 5. Conclusions

Comprehensive analysis indicates that water-saving irrigation contributes to reducing soil carbon emissions, while an appropriate proportion of organic fertilizer substitution reduces the carbon footprint and enhances the carbon sequestration capacity of the wheat field system by increasing crop carbon sequestration. Therefore, the suitable cropping pattern for wheat production in the Hetao Irrigation District is as follows: under the water-saving irrigation regime (irrigated twice at the jointing and heading stages, with an irrigation amount of 900 m^3^ ha^−1^ each time), apply 450 kg ha^−1^ of diammonium phosphate (DAP) as base fertilizer, and substitute urea with organic fertilizer at a proportion of 21.6–31.7% (with the organic fertilizer application rate being 1008.0 kg ha^−1^); additionally, top-dress 249.05 kg ha^−1^ of urea at the jointing stage. This cropping pattern can effectively balance the dual goals of water conservation and carbon emission reduction.

At present, the wheat photosynthetic carbon sequestration efficiency under organic fertilizer substitution for chemical nitrogen fertilizer has been significantly improved, but its impact on soil carbon pool fractions remains unclear, which is the key research direction for our next step. Meanwhile, the regulatory pathways of water-saving irrigation on soil carbon emissions also need to be further clarified.

## Figures and Tables

**Table 1:** Correlation coefficient between soil properties and CO_2_ emission rate.

Year	Soil Properties	SO	EM	TI	JO	HE	FL	FI	MA
2023	Temperature in 10 cm (°C)	0.125	0.419	0.657 *	0.693 *	0.742 *	0.815 **	0.961 **	0.728 *
	Temperature in 20 cm (°C)	0.106	0.372	0.401	0.610 *	0.684 *	0.897 **	0.816 **	0.701 *
	Water content in 10 cm (%)	0.071	0.612 *	0.764 *	0.673 *	0.750 *	0.805 **	0.741 *	0.629 *
	Compactness in 20 cm (Pa)	0.174	0.107	0.282	0.315	0.275	0.324	0.328	0.231
	Compactness in 40 cm (Pa)	0.221	0.197	0.348	0.486	0.425	0.298	0.352	0.429
	pH value in 20 cm	0.227	0.306	0.247	0.193	0.159	0.208	0.125	0.302
2024	Temperature in 10 cm (°C)	−0.590	0.020	0.284	0.650 **	0.365 *	0.494 **	0.368 *	0.450 *
	Temperature in 20 cm (°C)	−0.265	−0.340	0.298	0.391 *	0.365 *	0.518 *	0.485 **	0.419 *
	Water content in 10 cm (%)	0.010	0.349	0.320	0.972 **	0.363 *	0.364 *	0.361 *	0.418 *
	Compactness in 20 cm (Pa)	0.320	0.011	0.018	0.336	0.028	0.003	0.025	0.339
	Compactness in 40 cm (Pa)	0.251	0.022	0.358	0.057	0.090	0.046	0.107	0.110
	pH value in 20 cm	0.275	0.277	0.150	0.085	0.182	0.123	0.165	0.022

Value are the correlation coefficients between soil properties and CO_2_ emission rate. * and ** means correlation coefficient reach the significant level at *p* < 0.05 and *p* < 0.01. SO, EM, TI, JO, HE, FL, FI, MA represents the wheat growing period at sowing, emergence, tilling, jointing, heading, flowering, filling and mature, respectively.

**Table 2:** Carbon footprint composition under different treatments (kg ha^−1^).

Years	Treatment	Agricultural Production Materials CO_2_ Emissions						Soil Emissions	Total Emissions	Total Absorption	CO_2_ Budget NEP
		Seeds	CF	OF	IG	FM	PT				
2023	2WCK	239.3	1307.3	0	1107.0	26.6	15.2	3474.5	6169.9	7665.5 c	−1495.6
	2WT1	239.3	1163.8	66.8	1107.0	26.6	15.2	3578.5	6197.2	9440.7 a	−3243.5
	2WT2	239.3	1020.4	133.5	1107.0	26.6	15.2	3824.4	6366.4	8193.1 b	−1826.7
	2WT3	239.3	876.9	200.3	1107.0	26.6	15.2	3946.2	6411.5	7429.7 d	−1018.2
	2WT4	239.3	733.5	267.0	1107.0	26.6	15.2	4128.0	6516.6	7274.5 e	−757.9
	4WCK	239.3	1307.3	0	2214.0	26.6	15.2	3752.4	7554.8	8313.1 b	−758.3
	4WT1	239.3	1163.8	66.8	2214.0	26.6	15.2	3930.5	7656.2	10049.0 a	−2392.8
	4WT2	239.3	1020.4	133.5	2214.0	26.6	15.2	4154.9	7803.9	8654.5 b	−850.6
	4WT3	239.3	876.9	200.3	2214.0	26.6	15.2	4331.7	7904.0	7497.9 c	406.1
	4WT4	239.3	733.5	267.0	2214.0	26.6	15.2	4512.6	8008.2	7355.2 c	653.0
2024	2WCK	239.3	1307.3	0	1107.0	26.6	15.2	3204.7	5900.1	7974.4 c	−2074.3
	2WT1	239.3	1163.8	66.8	1107.0	26.6	15.2	3323.2	5941.9	8934.5 a	−2992.6
	2WT2	239.3	1020.4	133.5	1107.0	26.6	15.2	3478.5	6020.5	8407.6 b	−2387.1
	2WT3	239.3	876.9	200.3	1107.0	26.6	15.2	3590.8	6056.1	8008.0 c	−1951.9
	2WT4	239.3	733.5	267.0	1107.0	26.6	15.2	3778.8	6167.4	6325.8 d	−158.4
	4WCK	239.3	1307.3	0	2214.0	26.6	15.2	3555.0	7357.4	8491.6 c	−1134.2
	4WT1	239.3	1163.8	66.8	2214.0	26.6	15.2	3645.0	7370.7	9486.3 a	−2115.6
	4WT2	239.3	1020.4	133.5	2214.0	26.6	15.2	3840.2	7489.2	8935.1 b	−1445.9
	4WT3	239.3	876.9	200.3	2214.0	26.6	15.2	4002.4	7574.7	8301.0 c	−726.3
	4WT4	239.3	733.5	267.0	2214.0	26.6	15.2	4138.5	7634.1	6542.3 d	1091.8

CF: chemical fertilizers; OF: organic fertilizers; IG: irrigation; FM: fuel machinery; PT: pesticides. Total absorption is the carbon sequestration of the total wheat biomass. Total carbon emissions are the sum of carbon emissions from inputs to production materials and carbon emissions from wheat field soil. Net ecosystem productivity is the difference between total carbon absorption and total carbon emissions. Lowercase letters indicate significant differences among fertilization treatments (*p* < 0.05).

**Table 3:** Application of organic and chemical fertilizers in different treatments (kg ha^−1^).

Treatment Code	Starter Fertilizer		Top Dressing Urea (45% N)
	Organic Fertilizer	Diammonium Phos-Phate (18% N, 46% P_2_O_5_)	
2WCK	0	450	375.00
2WT1	750	450	281.25
2WT2	1500	450	187.50
2WT3	2250	450	93.75
2WT4	3000	450	0
4WCK	0	450	375.00
4WT1	750	450	281.25
4WT2	1500	450	187.50
4WT3	2250	450	93.75
4WT4	3000	450	0

**Table 4:** Carbon emission factors for various means of agricultural production.

Input Item	Emission Factor	Data Source
Nitrogen Fertilizer	1.53 kg kg^−1^	CLCD v0.8 database [38]
Phosphate Fertilizer	1.63 kg kg^−1^	CLCD v0.8 database
Potash Fertilizer	0.65 kg kg^−1^	CLCD v0.8 database
Organic Fertilizer	0.089 kg kg^−1^	CLCD v0.8 database
Herbicide	10.15 kg kg^−1^	Ecoinventv2.2 database [39]
Diesel Fuel	0.59 kg kg^−1^	IPCC Guidelines
Electricity	0.82 kg (kw h)^−1^	CLCD v0.8 database
Wheat Seeds	0.58 kg kg^−1^	Ecoinventv3.1 database [39]

## Data Availability

No new data were created or analyzed in this study.
